# Protein Engineering for Nicotinamide Coenzyme Specificity in Oxidoreductases: Attempts and Challenges

**DOI:** 10.3389/fmicb.2018.00194

**Published:** 2018-02-14

**Authors:** Andrea M. Chánique, Loreto P. Parra

**Affiliations:** ^1^Department of Chemical and Bioprocesses Engineering, School of Engineering, Pontificia Universidad Católica de Chile, Santiago, Chile; ^2^Schools of Engineering, Medicine and Biological Sciences, Institute for Biological and Medical Engineering, Pontificia Universidad Católica de Chile, Santiago, Chile

**Keywords:** oxidoreductases, coenzyme, enzyme engineering, cofactor, NAD(P)H

## Abstract

Oxidoreductases are ubiquitous enzymes that catalyze an extensive range of chemical reactions with great specificity, efficiency, and selectivity. Most oxidoreductases are nicotinamide cofactor-dependent enzymes with a strong preference for NADP or NAD. Because these coenzymes differ in stability, bioavailability and costs, the enzyme preference for a specific coenzyme is an important issue for practical applications. Different approaches for the manipulation of coenzyme specificity have been reported, with different degrees of success. Here we present various attempts for the switching of nicotinamide coenzyme preference in oxidoreductases by protein engineering. This review covers 103 enzyme engineering studies from 82 articles and evaluates the accomplishments in terms of coenzyme specificity and catalytic efficiency compared to wild type enzymes of different classes. We analyzed different protein engineering strategies and related them with the degree of success in inverting the cofactor specificity. In general, catalytic activity is compromised when coenzyme specificity is reversed, however when switching from NAD to NADP, better results are obtained. In most of the cases, rational strategies were used, predominantly with loop exchange generating the best results. In general, the tendency of removing acidic residues and incorporating basic residues is the strategy of choice when trying to change specificity from NAD to NADP, and *vice versa*. Computational strategies and algorithms are also covered as helpful tools to guide protein engineering strategies. This mini review aims to give a general introduction to the topic, giving an overview of tools and information to work in protein engineering for the reversal of coenzyme specificity.

## Introduction

Oxidoreductases (EC.1.X.X.X) are a large group of enzymes that catalyze the transfer of electrons from one molecule to another. These enzymes are valuable biocatalysts for industrial uses, since they allow the use of water as solvent and facilitate regio- stereo- and enantioselective conversions. Therefore, oxidoreductases have several applications in the chemical industry, mainly for the production of pharmaceuticals, agrochemicals, biofuels, polymers, amino acids, cosmetics, and nutraceuticals (May and Padgette, 1983; May, 1999; Xu, 2005).

Oxidoreductases usually need tightly bound cofactors for their catalytic activity, therefore forming part of the enzyme structure permanently as prosthetic groups. Oxidoreductases can also function with external electron donors or acceptors; in this case these cofactors are referred to as coenzymes (Torres Pazmiño et al., 2010) and stoichiometric amounts of them are required for the biotransformation at hand. Among oxidoreductases coenzymes, nicotinamide adenine dinucleotide (NAD) or its phosphorylated equivalent, nicotinamide adenine dinucleotide phosphate (NADP), are the most typical, either in their reduced and oxidized forms. These molecules possess two structural moieties; (i) the nicotinamide, giving the coenzyme its electrochemical function, accepting or donating a hydride group from the C-4 position (Paul et al., 2014) and, (ii) the adenosine, containing the phosphate group (NADP) or the hydroxyl group (NAD) in the 2'-position of the ribose giving the coenzyme its distinction (Knaus et al., 2016). Oxidoreductases are usually specific for one of the coenzymes, those involved in anabolic processes prefer NADP, and the ones participating in catabolic processes prefer NAD (Takase et al., 2014).

Different structural motifs enable the union of the coenzyme and give the specificity for NAD or NADP. Usually, enzymes preferring NADP have larger pockets with positively charged or hydrogen bond donating residues that interact with the phosphate group of the adenine ribose (Pick et al., 2014). NAD preferring enzymes contain negatively charged amino acids that generate repulsion toward NADP and form hydrogen bonds to the 2′-OH and 3′-OH of the adenine ribose (Petschacher et al., 2014). A recurring structural motif for the binding of coenzymes is the Rossman fold. This nucleotide-binding motif is formed by two α-helices and three β-strands in the alternating pattern βαβαβ. The pyrophosphate union site is located at the amino terminus of the first α-helix, characterized by the conserved sequence GxGxxG in NAD dependent enzymes and GxGxxA in NADP dependent ones (Hanukoglu and Gutfinger, 1989). The specificity to each coenzyme is influenced by the C-terminus of the second β-strand, where an acidic residue is usually present for NAD preferring enzymes (Carugo and Argos, 1997). Another coenzyme binding fold is the TIM barrel. This conformation can, among others, be observed in the aldo-keto reductase superfamily and is formed by eight α-helices and eight β-strands, alternating and forming a barrel with the β-strands in the interior and the α-helix in the exterior (Solanki et al., 2017).

Switching the coenzyme preference in oxidoreductases is an attractive research area, particularly when these enzymes are used in biocatalysis and metabolic engineering. In some cases, the enzyme is part of a cascade that allows intelligent use of an alternative coenzyme (Gand et al., 2016). The bioavailability of one of the coenzymes or the easiness of their regeneration has been another topic of research (Lerchner et al., 2016). In cell free biotransformations, NAD is usually preferred over NADP due to its much lower price and higher stability (Beier et al., 2016). For some applications it can be desirable that the enzyme uses both coenzymes to increase process efficiency and circumvent metabolic bottlenecks (Pick et al., 2014; Solanki et al., 2016).

In this mini review we focus on the achievements to change the coenzyme preference of oxidoreductases reviewing various coenzyme engineering attempts. We analyzed the different protein engineering strategies and related them with the degree of success in switching the cofactor preference. Computational strategies and algorithms are also covered as helpful complements for the guidance of protein engineering.

## Attempts to change the coenzyme specificity in oxidoreductases

We reviewed 103 enzymes that have been engineered considering their coenzyme specificity and updated the data provided by Cahn et al. (2017) to build a corresponding table (Table 1). To evaluate the degree of success of the reported results, we used three parameters: (i) Coenzyme Specificity Ratio, reflecting the degree of preference of the target coenzyme in the mutated enzyme (Equation 1 when switching from NAD to NADP, and Equation 2 for the opposite direction), (ii) Relative Catalytic Efficiency, which compares the catalytic efficiency of the mutated enzyme with the desired coenzyme and the wild type enzyme using its natural coenzyme (Equation 3 when switching from NAD to NADP, and Equation 4 for the opposite direction), and (iii) Relative Specificity, which compares the coenzyme specificity between the mutated and wildtype enzymes (Equation 5 when switching from NAD to NADP, and Equation 6 for the opposite direction) (Cahn et al., 2017).

(1) $$\begin{array}{r} \frac{{\left(\frac{k_{cat}}{K_{m}}\right)}_{NADP}}{{\left(\frac{k_{cat}}{K_{m}}\right)}_{NAD}} \end{array}$$

(2) $$\begin{array}{r} \frac{{\left(\frac{k_{cat}}{K_{m}}\right)}_{NAD}}{{\left(\frac{k_{cat}}{K_{m}}\right)}_{NADP}} \end{array}$$

(3) $$\begin{array}{r} \frac{{\left(\frac{k_{cat}}{K_{m}}\right)}_{NADP}^{mut}}{{\left(\frac{k_{cat}}{K_{m}}\right)}_{NAD}^{WT}} \end{array}$$

(4) $$\begin{array}{r} \frac{{\left(\frac{k_{cat}}{K_{m}}\right)}_{NAD}^{mut}}{{\left(\frac{k_{cat}}{K_{m}}\right)}_{NADP}^{WT}} \end{array}$$

(5) $$\begin{array}{r} \frac{{\left(\frac{{\left(\frac{k_{cat}}{K_{m}}\right)}_{NADP}}{{\left(\frac{k_{cat}}{K_{m}}\right)}_{NAD}}\right)}^{mut}}{{\left(\frac{{\left(\frac{k_{cat}}{K_{m}}\right)}_{NADP}}{{\left(\frac{k_{cat}}{K_{m}}\right)}_{NAD}}\right)}^{WT}} \end{array}$$

(6) $$\begin{array}{r} \frac{{\left(\frac{{\left(\frac{k_{cat}}{K_{m}}\right)}_{NAD}}{{\left(\frac{k_{cat}}{K_{m}}\right)}_{NADP}}\right)}^{mut}}{{\left(\frac{{\left(\frac{k_{cat}}{K_{m}}\right)}_{NAD}}{{\left(\frac{k_{cat}}{K_{m}}\right)}_{NADP}}\right)}^{WT}} \end{array}$$

**Table 1:** Reviewed oxidoreductases subjected to coenzyme engineering.

**Enzyme**	**Site directed**	**Saturation**	**Loop exchange**	**Computational**	**MSA**	**Bibliography**	**Structural**	**Mutations**	**Reaction^a^**	**CSR^b^**	**RCE^c^**	**log RS^d^**	**Source**
**EC 1.1. FROM NAD TO NADP**													
L-Arabinitol 4-dehydrogenase (*N. crassa*)	^*^	^*^			^*^			D211S. I212R. S348T	Ox	21.900	0.462	3.539	Bae et al., 2010
D-(-)- lactate dehydrogenase (*L. delbruckii*)	^*^				^*^		^*^	D175A	Red	1.000	0.110	5.041	Bernard et al., 1995
Mannitol 2-dehydrogenase (*P. fluorescens*)	^*^						^*^	E68K.D69A	Red	18.667	5.833	4.651	Bubner et al., 2008
Alcohol dehydrogenase (*Drosophila*)	^*^					^*^		D39N	Ox	0.648	0.638	2.778	Chen et al., 1994
Alcohol dehydrogenase (*Drosophila*)	^*^					^*^		D39N. A46R	Ox	1.000	0.272	2.967	Chen et al., 1994
Isopropylmalate dehydrogenase (*T. thermophilus*)	^*^		^*^				^*^	–	Ox	1000.000	1.549	4.940	Chen et al., 1996
Polyol dehydrogenase (*G. oxydans*)	^*^			^*^				Q20R. D43S	Red	1.500	1.175	NA	Cui et al., 2015
Xylitol dehydrogenase (*G. oxydans*)	^*^				^*^		^*^	D38S. M39R	Ox	0.900	1.250	NA	Ehrensberger et al., 2006
2,3-butanediol dehydrogenase (*S. cerevisae*)	^*^						^*^	E221S. I222R. A223S	Red	0.900	1.148	NA	Ehsani et al., 2009
Lactate dehydrogenase (*B. stearothermophilus*)	^*^						^*^	D53S	Red	0.150	0.050	0.531	Feeney et al., 1990
3-hydroxy-3-methylglutaryl coenzyme A (*P. mevalonii*)	^*^				^*^		^*^	D146A. L148K	Ox	0.140	2.*E*−04	4.881	Friesen et al., 1996
Malate dehydrogenase (*S. coelicolor*)	^*^				^*^			E42G. I43S. P45R. A46S	Red	10.250	1.938	3.351	Ge et al., 2014
Lactate dehydrogenase (*B. stearothermophilus*)	^*^					^*^		I51K. D52S	Red	2.200	0.025	1.690	Holmberg et al., 1999
Mitochondrial malic enzyme (*H. sapiens*)	^*^				^*^	^*^		Q362K	Ox	3.200	0.295	2.462	Hsieh et al., 2006
L-arabinitol dehydrogenase (*A. niger*)	^*^				^*^			D213S. I214R. 359T	Ox	100.000	0.051	4.101	Kim et al., 2010
L-arabinitol dehydrogenases (*T. longibrachiatum*)	^*^				^*^			D224S. I225R. A362T	Ox	161.034	0.347	4.406	Kim et al., 2010
L-arabinitol dehydrogenases (*P. chrysogenum*)	^*^				^*^			D212S. I213R. S358T	Ox	NA	0.001	NA	Kim et al., 2010
1,3-propanediol oxidoreductase (*K. pneumoniae*)	^*^							D41G	Red	1.560	0.219	NA	Ma et al., 2010
Isopropylmalate dehydrogenase (*E. coli*)	^*^						^*^	K100R. A229T. D236R. L248M. D289K. I290Y. A296V. G337Y	Ox	370.000	0.589	4.708	Miller et al., 2006
Malate Dehydrogenase (*T. flavus*)			^*^					E41G. I42S. P43E. Q44R. A45S. M46F. K47Q	Red	24.000	0.339	2.716	Nishiyama et al., 1993
Alcohol Dehydrogenase (*P. furiosus*)	^*^					^*^		K249G. H255R	Ox	1.818	3.750	−0.314	Solanki et al., 2016
4-deoxy-L-erythro-5-hexoseulose uronate reductase (*Sphingomonas* sp.)			^*^					T16S. E17Q. N37H. S38G. H39R. V40K. D41A	Red	85.000	4.677	3.041	Takase et al., 2014
Xylitol dehydrogenase (*P. stipitis*)	^*^				^*^		^*^	D207A. I208R. F209T	Ox	2.600	1.122	4.041	Watanabe et al., 2005
Myo-Inositol dehydrogenase (*B. subtilis*)	^*^					^*^		A12K. D35S. V36R	Ox	4.800	1.072	NA	Zheng et al., 2013
**EC 1.1. FROM NADP TO NAD**													
Ketol-Acid Reductoisomerase *(E. coli*)		^*^					^*^	A71S. R76D. S78D. Q110V	Red	190.000	0.851	4.732	Bastian et al., 2011
Ketol-Acid Reductoisomerase (*A. acidocaldarius*)		^*^		^*^				R48P. S51L. S52D. R84A	Red	110.000	0.032	2.653	Brinkmann-Chen et al., 2013
Ketol-Acid Reductoisomerase (*M. aeolicus*)		^*^		^*^				G50D. S52D	Red	120.000	0.145	3.079	Brinkmann-Chen et al., 2013
Ketol-Acid Reductoisomerase (*S. exigua*)		^*^		^*^				S61D. S63D. I95V	Red	88.000	0.025	3.892	Brinkmann-Chen et al., 2013
Ketol-Acid Reductoisomerase (*L. lactis*)		^*^		^*^				V48L. R49P. K52L. S53D. E59K. T182S. E320K	Red	150.000	1.096	4.362	Brinkmann-Chen et al., 2013
Ketol-Acid Reductoisomerase (*Shewanella* sp.)		^*^		^*^				A71S. R76D. S78D. Q110V	Red	64.000	0.008	5.041	Brinkmann-Chen et al., 2013
Glyoxylate reductase (*A. thaliana*)		^*^		^*^				R31L. T32K. K35D. C68R	Red	2.400	0.195	1.519	Cahn et al., 2017
Cinnamyl alcohol dehydrogenase (*S. cerevisae*)		^*^		^*^				S210D. R211P. K215E. S253P	Red	2.000	5.888	1.820	Cahn et al., 2017
Iron-containing alcohol dehydrogenase (*T. maritima*)		^*^		^*^				G36E. S38N. S39G	Red	43.000	2.951	1.924	Cahn et al., 2017
Xylose reductase (*T. emersonii*)		^*^		^*^				S272G. N273G. R277Y. Q280E	Red	5.200	0.032	3.681	Cahn et al., 2017
Isocitrate Dehydrogenase (*E. coli*)	^*^					^*^	^*^	C201I. C332Y. K344D. Y345I. V351A. Y391K. R395S	Ox	200.000	0.032	6.146	Chen et al., 1995
1,5-anhydro-d-fructose reductase (*S. morelense*)	^*^					^*^	^*^	A13G. S33D	Red	14.000	0.162	NA	Dambe et al., 2006
Xylose reductase (*T. emersonii*)	^*^					^*^		K271R. N273D	Red	0.618	0.057	1.180	Fernandes et al., 2009
17 -hydroxysteroid dehydrogenase (*H. sapiens*)	^*^					^*^	^*^	E282A	Ox	0.175	0.206	0.076	Huang et al., 2001
Isocitrate Dehydrogenase (*E. coli*)	^*^				^*^		^*^	K344D. Y345I. V351A. Y391K. R395S. C332Y. C201M	Ox	202.469	0.035	6.140	Hurley et al., 1996
γ-Diketone Reductase (*S. cerevisiae*)	^*^					^*^	^*^	N9E	Red	0.856	0.026	1.963	Katzberg et al., 2010
Xylose reductase (*C. boidinii*)	^*^			^*^				K272G. S273G. N274D	Red	0.900	0.079	NA	Khoury et al., 2009
17β-hydroxysteroid dehydrogenase (*C. lunatus*)	^*^				^*^		^*^	Y49D	Ox	7.800	6.*E*−05	NA	Kristan et al., 2007
Xylose reductase (P. stipitis)		^*^		^*^			^*^	K270R. N272D	Red	2.900	0.063	0.968	Liang et al., 2007
Clostridial alcohol dehydrogenase (*C. autoethanogenum*)	^*^	^*^					^*^	G198D. S199V. P201E. Y218A	Red	0.900	0.000	NA	Maddock et al., 2015
Carbonyl reductase (*M. musculus*)	^*^						^*^	T38D	Red	31.000	0.309	3.114	Nakanishi et al., 1997
Xylose reductase (*C. tenuis*)	^*^					^*^	^*^	K274R. N276D	Red	1.200	0.063	1.279	Petschacher et al., 2005
Zinc-dependent alcohol dehydrogenase (*E. coli*)		^*^					^*^	S200N	Red	0.573	0.168	1.502	Pick et al., 2014
Zinc-dependent alcohol dehydrogenase (*E. coli*)		^*^					^*^	T?D. T?I. S?N	Red	2.188	1.000	1.583	Pick et al., 2014
Zinc-dependent alcohol dehydrogenase (*E. coli*)		^*^					^*^	S199C. S200N. N201D	Red	0.882	0.239	1.689	Pick et al., 2014
Glucose dehydrogenase (*H. mediterranei*)	^*^					^*^	^*^	G206D	Ox	116.667	0.078	3.378	Pire et al., 2009
Ketol-Acid Reductoisomerase (*E. coli*)	^*^				^*^			R68D. K69L. K75V. R76D	Red	31.000	0.282	4.763	Rane and Calvo, 1997
Isocitrate dehydrogenase (*H. volcanii*)	^*^				^*^	^*^		R291S. K343D. Y344I. V350A. Y390P	Ox	0.900	0.170	NA	Rodríguez-Arnedo et al., 2005
Alcohol dehydrogenase (*R. perezi*)	^*^						^*^	G223D. T224I. H225N	Ox	7600.000	3.548	4.908	Rosell et al., 2003
Malate dehydrogenase (*S. bicolor*)	^*^				^*^	^*^		G84D. S85I. R87Q. S88A	Red	12.000	0.110	4.322	Schepens et al., 2000
4-deoxy-L-erythro-5-hexoseulose uronate reductase (*Sphingomonas* sp.)			^*^					H37N. G38S. R39H. K40V. A41D	Red	0.900	0.001	NA	Takase et al., 2014
Isocitrate Dehydrogenase (*T. thermophilus*)	^*^		^*^		^*^	^*^		R231A. K283D. Y284I. N287G. V288I. I290A	Ox	68.000	0.079	4.716	Yaoi et al., 1996
Xylose reductase (*P. stipitis*)	^*^					^*^	^*^	K21A. N272D	Red	0.900	0.794	NA	Zeng et al., 2009
Short-chain carbonyl reductase (*C. parapsilosis*)	^*^							S67D. P69D	Red	0.310	0.661	0.663	Zhang et al., 2009
**EC 1.2. FROM NAD TO NADP**													
Formate dehydrogenase (*C. boidinii*)		^*^			^*^		^*^	D195Q. Y196H	Ox	0.963	0.001	8.355	Andreadeli et al., 2008
Glyceraldehyde3-phosphate dehydrogenase (*C. glutamicum*)	^*^						^*^	D35G. L36T. T37K. P192S	Ox	3.578	0.728	NA	Bommareddy et al., 2014
Glyceraldehyde3-phosphate dehydrogenase (*C. glutamicum*)	^*^						^*^	D35G. L36T. T37K	Ox	8.955	0.595	NA	Bommareddy et al., 2014
Glyceraldehyde-3-phosphate dehydrogenase (*B. stearothermophilus*)	^*^				^*^			D32A. L187A. P188S	Ox	1.600	0.020	NA	Clermont et al., 1993
Formate dehydrogenase (*C. methylica*)	^*^					^*^	^*^	D195S	Ox	0.024	0.033	4.785	Gul-karaguler et al., 2001
Formate dehydrogenase (*M. vaccae*)	^*^					^*^		C145S. A198G. D221Q. C225V	Ox	16.627	NA	NA	Hoelsch et al., 2013
Aldehyde dehydrogenase (*B. cereus*)	^*^				^*^			E194S	Ox	0.470	0.347	1.322	Hong et al., 2016
Formate dehydrogenase (*Pseudomonas* sp.)	^*^						^*^	-	Ox	3.500	0.100	NA	Serov et al., 2002
Formate dehydrogenase (*S. cerevisiae*)	^*^						^*^	D196A. Y197R	Ox	2.300	2.*E*−04	NA	Serov et al., 2002
Aldehyde dehydrogenase (*A. thaliana*)	^*^				^*^		^*^	E149T. V178R. I200V	Ox	6.854	0.804	NA	Stiti et al., 2014
Aldehyde dehydrogenase (*A. thaliana*)	^*^				^*^		^*^	E149T. I200V	Ox	0.794	0.184	NA	Stiti et al., 2014
Formate dehydrogenase (*C. bodinii*)		^*^				^*^		D195Q. Y196R. Q197N	Ox	17.118	0.436	6.058	Wu et al., 2009
**EC 1.2. FROM NADP TO NAD**													
Formate dehydrogenase (*B. stabilis*)	^*^				^*^	^*^		Q223E	Ox	125.000	0.667	3.510	Hatrongjit and Packdibamrung, 2010
Aldhehyde dehydrogenase (*V. harveyi*)	^*^				^*^			T175E	Ox	130.000	0.174	3.681	Zhang et al., 1999
**EC 1.4. FROM NAD TO NADP**													
Alanine dehydrogenase (*Shewanella* sp.)	^*^				^*^		^*^	D198A	Ox	14.000	0.110	NA	Ashida et al., 2004
Glutamate dehydrogenase (*C. symbiosum*)	^*^						^*^	F238S. P262S	Red	0.320	0.001	1.763	Capone et al., 2011
Glutamate dehydrogenase (*P. asaccharolyticus*)	^*^				^*^			E243K	Red	0.952	0.004	3.045	Carrigan and Engel, 2007
Leucine dehydrogenase (*T. intermedius*)	^*^				^*^			D203A. I204R. D210R	Ox	74.000	0.025	NA	Galkin et al., 1997
L-alanine dehydrogenase (*B. subtilis*)	^*^					^*^	^*^	D196A. L197R	Red	18.033	0.903	2.857	Lerchner et al., 2016
Glutamate dehydrogenase (*C. symbiosum*)	^*^				^*^		^*^	F238S. P262S. D263K. N290G	Ox	21.214	0.819	4.338	Sharkey et al., 2012
**EC 1.5. FROM NADP TO NAD**													
Imine reductase (*Streptomyces* sp.)	^*^				^*^		^*^	S37V. K40A	Red	5.500	0.025	2.255	Gand et al., 2016
F420: NADPH oxidoreductase (*T. fusca*)	^*^					^*^	^*^	R55S	Red	19.268	1.756	1.734	Kumar et al., 2017
**EC 1.6. FROM NAD TO NADP**													
Dihydrolipoamide dehydrogenase (*E. coli*)	^*^				^*^	^*^		E205V. M206R. F207K. D208H. P212R		0.900	4.677	NA	Bocanegra et al., 1993
Cytochrome b5 reductase (*R. norvegicus*)	^*^				^*^	^*^		D239T	Red	10.000	0.214	4.613	Marohnic et al., 2003
NAD(P)H oxidase (*S. mutans*)	^*^						^*^	V193R. V194H	Red	1.000	0.600	4.699	Petschacher et al., 2014
NAD(P)H oxidase (*S. mutans*)	^*^						^*^	D192A. V193R. V194H. A199R	Red	10.000	3.631	4.806	Petschacher et al., 2014
Cytochrome P450 reductase (*H. sapiens*)	^*^					^*^		W676H	Red	0.002	0.003	0.908	Döhr et al., 2001
Cytochrome P450 reductase (*H. sapiens*)	^*^					^*^		W676A	Red	0.236	0.045	3.013	Döhr et al., 2001
P450R (*R. norvegicus*)	^*^					^*^		W677A	Red	1.156	0.016	4.724	Elmore and Porter, 2002
Glutathione reductase (*E. coli*)	^*^					^*^	^*^	A179G. A183G. V197E. R198M. K199F. H200D. R204P	Ox	8.133	0.033	4.248	Scrutton et al., 1990
**EC 1.6. FROM NADP TO NAD**													
Nitrate reductase (*N. crassa*)	^*^						^*^	S920D. R932S	Red	65.000	0.003	4.857	Shiraishi et al., 1998
**EC 1.14. FROM NAD TO NADP**													
Flavoprotein monooxygenase (*S. maltophilia*)	^*^						^*^	H194T	Red	3.501	2.453	0.726	Jensen et al., 2013
**EC 1.14. FROM NADP TO NAD**													
Cyclohexanone monooxygenase (*Acinetobacter* sp.)	^*^				^*^	^*^	^*^	S186P. S208E. K326H	Red	4.727	0.001	4.404	Beier et al., 2016
Phenylacetone monooxygenase (*T. fusca*)	^*^				^*^		^*^	H220Q	Red	0.001	0.001	0.915	Dudek et al., 2010
p-Hydroxybenzoate hydroxylase (*P. fluorescens*)	^*^						^*^	R33S. Q34R. P36R. D37A. Y38E	Red	3.120	0.010	4.703	Eppink et al., 1999
P450 PMO R2 (*B. megaterium*)	^*^					^*^		R966N. K972H. Y974F. W1046D	Red	0.620	0.079	2.643	Fasan et al., 2011
Flavocytochrome P450 BM3 (*B. megaterium*)	^*^					^*^		W1046A	Red	0.629	0.038	2.331	Girvan et al., 2011
Cyclohexanone monooxygenase (*Acinetobacter* sp.)	^*^	^*^			^*^			K326A	Red	0.096	0.001	1.741	Kamerbeek et al., 2004
4-hydroxyacetophenone monooxygenase (P. fluorescens)	^*^	^*^			^*^			K439N	Red	0.156	0.008	2.037	Kamerbeek et al., 2004
4-hydroxyacetophenone monooxygenase (*P. fluorescens*)	^*^	^*^			^*^			K439F	Red	0.615	0.010	2.634	Kamerbeek et al., 2004
Flavocytochrome P450 BM3 (*B. megaterium*)	^*^				^*^	^*^		R966D. W1046S	Red	0.370	0.398	2.362	Maurer et al., 2005
**EC 1.18. FROM NADP TO NAD**													
Ferredoxin-NADP (+) reductase (*P. falciparum*)	^*^					^*^	^*^	Y258F	Red	0.670	0.063	1.672	Baroni et al., 2012
Ferredoxin-NADP+ Reductase (*Anabaena PCC7119*)	^*^				^*^		^*^	S223D	Red	0.120	4.*E*−06	3.908	Medina et al., 2001
Ferredoxin-NADP(H) reductase (*P. sativum*)	^*^					^*^		Y308S	Ox	0.020	0.025	2.519	Paladini et al., 2009
**EC 1.20 FROM NAD TO NADP**													
Phosphite Dehydrogenase (*P. stutzeri*)	^*^					^*^	^*^	E175A. A176R	Ox	2.800	10.000	2.477	Woodyer et al., 2003

*NA, Not available*.

a *As used in the paper for the essays. For multistep enzymes, it was considered the step in which the cofactor gives or accepts the proton*.

b *CSR, Coenzyme Specificity Ratio*.

c *RCE, Relative Catalytic Efficiency*.

d *log RS, Logarithm of the Relative Specificity*.

**Figure 1:**
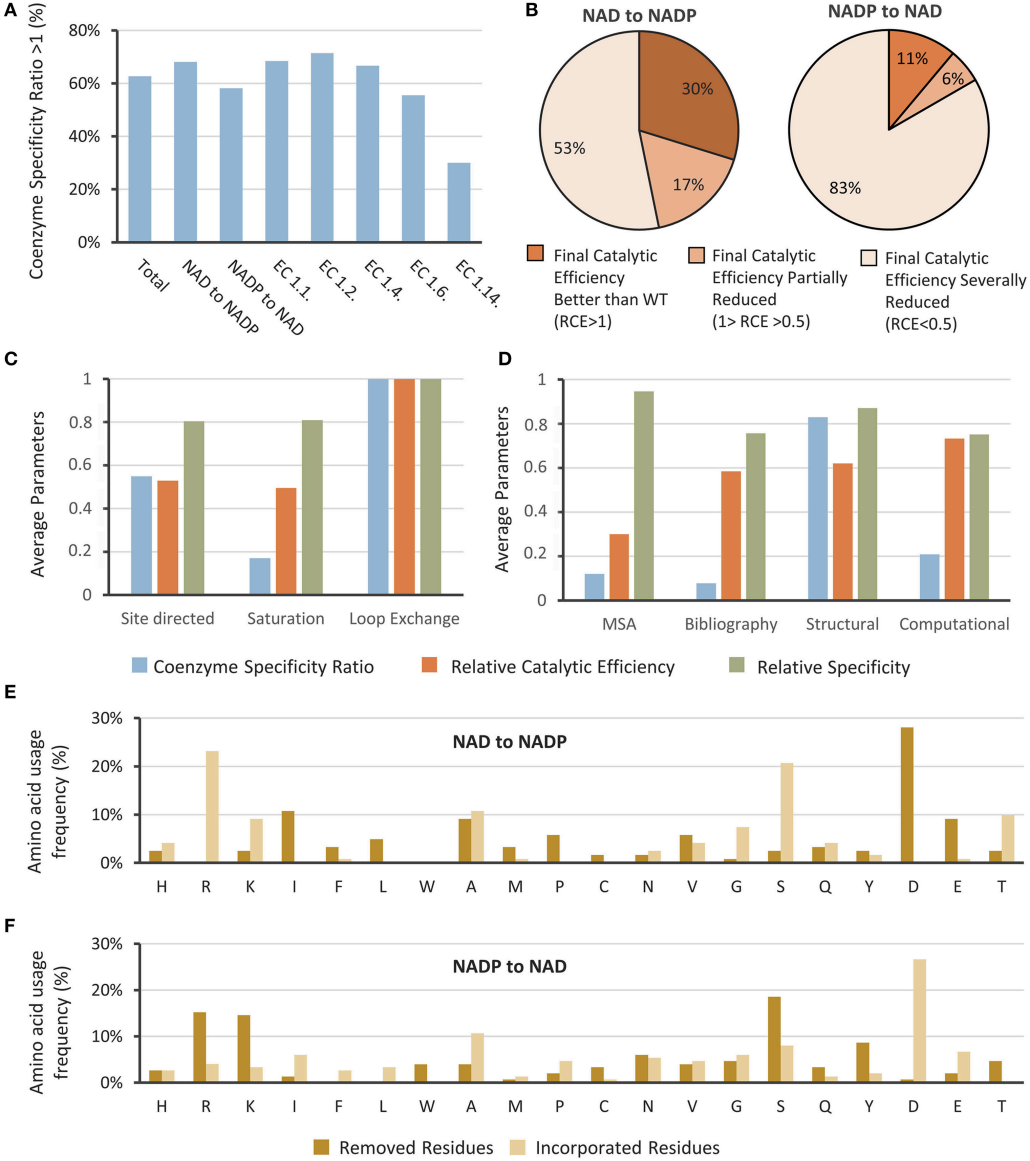
Attempts to change the coenzyme preference in oxidoreductases. **(A)** Studies where coenzyme specificity was successfully reversed were analyzed by their Coenzyme Specificity and classified by the EC number and according to the target coenzyme. **(B)** Achievements were analyzed regarding their Relative Catalytic Efficiency. Normalized values for the averages of Coenzyme Specificity, Relative Catalytic Efficiency, and Relative Specificity classified according to **(C)** the protein engineering and **(D)** mutagenesis strategies employed. Frequency of the incorporation or removal of amino acids when changing specificity from **(E)** NAD to NADP and from **(F)** NAD to NADP.

We classified the attempts by the EC number, and analyzed the data regarding the degree of accomplishment in switching the cofactor specificity. Different parameters were taken into account, which are represented in several graphics shown in Figure 1.

We could observe that in 62% of the cases the Coenzyme Specificity Ratio was greater than 1 (Figure 1A), meaning that the coenzyme preference was reversed. Despite the fact that 38% of the attempts to switch the enzymes' cofactor resulted in Coenzyme Specificities Ratios below one, in some cases the research goal was to obtain an enzyme that functions with both cofactors; therefore a Coenzyme Specificity Ratio close to one should be considered a satisfactory result (Woodyer et al., 2003; Petschacher et al., 2014).

Most of the studies were performed in enzymes belonging to EC 1.1, with 58 cases considered in this review. The best results were obtained for oxidoreductases of the aforementioned class, together with the ones belonging to EC 1.2. Enzymes classified in EC 1.6 and 1.14 gave poor results. Most enzymes belonging to EC 1.14 are NADP-dependent Baeyer-Villiger monooxigenases, it has been reported that switching the coenzyme preference in these is more challenging, due to the complexity of their electron transfer mechanisms (Beier et al., 2016; Cahn et al., 2017). Recently, Beier et al. (2016) switched the coenzyme preference of a Baeyer-Villiger monooxygenase. The best variant showed higher conversion of cyclohexanone with NADH than using NADPH, however the Coenzyme Specificity Ratio and Relative Catalytic Efficiency were 4.7 and 0.0015 respectively.

Regarding the Relative Catalytic Efficiency, results were separated in three scenarios: (i) Higher than one, indicating that the catalytic efficiency of the mutated enzyme with the desired coenzyme was better than the catalytic efficiency of the wild type enzyme with its natural coenzyme, (ii) Lower than one but higher than 0.5, indicating that the catalytic efficiency was reduced by less than 50%, and (iii) Lower than 0.5, which reflects an important reduction of the enzyme functionality. Figure 1B shows the results analyzed by this index when changing from NADP to NAD and vice versa. Most of the attempts resulted in variants with decreased Relative Catalytic Efficiency using the targeted coenzyme. When trying to alter the coenzyme specificity from NAD to NADP, only in 30% of the cases the catalytic efficiency of the obtained variant with NADP was better than the catalytic efficiency of the wild type enzyme with NAD. When changing from NADP to NAD, only 11% of the cases showed better catalytic efficiencies, meaning that 89% of the variants showed a reduced catalytic efficiency when using NAD. Altogether these results show that a change in coenzyme specificity typically leads to a loss of functionality of the enzyme, as has been reported before (Cahn et al., 2017).

The Relative Specificity was also calculated and a log-transformation (base 10) was applied for better analysis of the data (Table 1). Only one of the studies reported an enzyme with values lower than 1 (Solanki et al., 2016). The rest of the values ranged from 1.1 to 8.4, indicating a favorable change in cofactor preference. The lowest values were obtained when changing from NADP to NAD, averaging a value of 3.0, as compared to NAD to NADP changes which averaged 3.5. Enzymes belonging to EC 1.2 averaged the best value for this parameter (4.6), followed by EC 1.6 (4.0) and EC 1.1 (3.1). Enzymes from EC 1.14 averaged the lowest values (2.5). Although the Relative Specificity gives information regarding the reversal success, it does not indicate information about the absolute degree of cofactor preference or usability of the new enzyme.

The protein engineering approaches utilized were rational or semi-rational, and we grouped them in three categories: site directed mutagenesis, saturation mutagenesis and loop exchange (Figure 1C). Site directed mutagenesis was employed in 82 opportunities, saturation mutagenesis in 20 opportunities, and loop exchange was used in 5 cases and gave best results. In this strategy, a complete region determining cofactor specificity is replaced for a region of another enzyme reported to have the desired cofactor specificity. This approach was used in a DEH reductase (Takase et al., 2014) where two loops were exchanged to switch the specificity from NADH to NADPH, giving excellent results. Despite these good achievements, only a few studies have been reported using loop exchange. One of the limitations of this strategy is to find proper coenzyme-binding loops in a protein with a highly structural identity with the target enzyme.

As we have noticed, rational design is the preferred strategy used for coenzyme engineering; and strategic positions within the cofactor binding site should be identified. We were curious about the criteria used for selection of the residues, and also evaluated the rate of success regarding this parameter (Figure 1D). We classified the approaches in 4 categories: (i) multiple sequence alignments (MSA, 37 cases), (ii) rational transfer of amino acids of previously reported studies (Bibliography, 36 cases), (iii) structural analysis of the coenzyme binding site (Structure, 51 cases), and (iv) computational approaches and use of algorithms (Computational, 12 cases). Some works employed more than one of these approaches, therefore they were considered in each case. As the analysis suggest (Figure 1D), when examining the coenzyme binding site good results were obtained. Therefore, we strongly recommend using this approach for a proper selection of the target positions. MSA and bibliography could also be used as a complement. When computational tools or algorithms were used, their average Relative Catalytic Efficiency was the highest (Figure 1D), therefore we evaluate this approach separately.

We also evaluated the frequencies in amino acids that were removed or incorporated for the engineering of coenzyme specificity. Usually, acidic residues are mutated to switch coenzyme specificity from NAD to NADP (Figure 1E), and are used as a replacement in the opposite case (Figure 1F). In the analyzed studies, when switching the coenzyme specificity from NAD to NADP, 28% of the residues corresponded to Asp and 9% to Glu, covering together over one third of the mutations (Figure 1E). When seeking the switch from NADP to NAD, Asp and Glu were added in 34% of the cases (Figure 1F). The relevance of these residues has been reported in other occasions (Brinkmann-Chen et al., 2014; Pick et al., 2014), and is mainly due to the acidic groups repulsion with the negatively charged phosphate of the NADP (Jensen et al., 2014). On the other hand, amino acids with positive charges stabilize the binding of NADP. Actually, in NADP binding enzymes containing a Rossmann fold, an Arg forms a cation-pi interaction with the adenine ring system (Cahn et al., 2017). Therefore, it is not unexpected that Arg and Lys were the most incorporated amino acid when switching from NAD to NADP, and most replaced ones when inverting the cofactor specificity to NAD (Schepens et al., 2000; Brinkmann-Chen et al., 2013).

Alanine has also been frequently used for both, as a target residue and a replacement for bidirectional coenzyme engineering (Figures 1E,F). In some cases, alanine has been used to remove a hydrogen bond and debilitate the interaction between the natural coenzyme and its binding site (Woodyer et al., 2003; Bubner et al., 2008; Zeng et al., 2009; Lerchner et al., 2016). In other studies, Ala was used to increase the flexibility and size of the coenzyme binding site for a better acceptance of NADP (Hoelsch et al., 2013). Among these lines, alanine has also been used to replace the third glycine of the NADH-binding motif GxGxxG, hampering the interactions occurring in the Rossman fold and facilitating the use of NADP (Dambe et al., 2006). Alanine was also applied for the switching from NADP to NAD, this was the case for an isocitrate dehydrogenase where a valine was mutated to alanine reducing the distance between an Asp and the 2'- and 3'- hydroxyls of the ribose (Rodríguez-Arnedo et al., 2005). When switching from NADP to NAD, alanine was used to replace larger and more acidic amino acids, such as aspartate or glutamate, to broaden the binding site and facilitate the union of NADP (Zheng et al., 2013). Moreover, alanine scanning has been used to study the relevance of some positions for coenzyme binding (Kamerbeek et al., 2004).

Serine has been targeted to change coenzyme specificity toward NAD, and incorporated in the other direction (Figures 1E,F). Ser usually interacts with the phosphate group of NADP stabilizing the coenzyme binding (Schepens et al., 2000; Ge et al., 2014). The short side chain of Ser makes it difficult for the OH groups of NAD-adenine moiety to interact with this residue in the coenzyme binding site (Ge et al., 2014). Therefore, in several studies a serine has been replaced to switch from NADP to NAD usage (Medina et al., 2001; Khoury et al., 2009), by Asp (Bastian et al., 2011; Brinkmann-Chen et al., 2013) and Arg (Chen et al., 1995; Rodríguez-Arnedo et al., 2005) respectively.

## Computational tools and algorithms for switching the coenzyme preference

Recently, computational tools and algorithms have been applied to assist in the selection of “hot” positions for coenzyme engineering. Cui et al. (2015) proposed a computational approach that enhances the hydrogen-bond interaction between an enzyme and its coenzyme, using only the protein structure of the target protein. Using this strategy, they reversed the coenzyme specificity of a dehydrogenase from NADH to NADPH (Cui et al., 2015). Khoury et al. (2009) used a computational approach based on the iterative protein redesign and optimization algorithm (IPRO). With this algorithm they generated *in silico* mutations to improve binding of NADH to the target enzyme evidenced by improved interaction energies. Seven out of ten designed mutants showed a significant switch in coenzyme specificity toward the desired coenzyme and two showed dual coenzyme specificity (Khoury et al., 2009). Brinkmann-Chen et al. (2014) developed an algorithm to reverse the cofactor preference based on structural analysis of the enzymes.

Cahn et al. (2017) developed a web tool for switching coenzyme preference in a general approach, allowing for its application to any oxidoreductase. This structure-guided, semi rational strategy named SCR-SALAD (Coenzyme Specificity Reversal–Structural Analysis and Library Design) involves three steps: (i) analysis of enzyme structure to detect crucial residues determining coenzyme specificity, (ii) design of small degenerated codon libraries targeting the detected positions and, (iii) recovery of the catalytic efficiency which is usually lost during modification. Using this program, cofactor specificity was efficiently switched in four structurally diverse NADP-dependent enzymes. Despite the fact, that the authors did not try to reverse the coenzyme specificity of NAD-dependent enzymes, comparison of previously published studies with CSR-SALAD showed, that the generated libraries contained all the beneficial mutations for reversing the specificity from NAD to NADP. Although the results obtained by using CSR-SALAD are promising, this web tool has not proven useful in multistep electron transfer pathways (for instance mono- and dioxygenases) and does not consider natural evolution with insertions and deletions (Cahn et al., 2017).

## Conclusions

Efforts on inverting the cofactor specificity of oxidoreductases have been made for practical reasons. The process has proven to be complex, even though a reversal of the preference is usually achieved, a loss of efficiency regularly appears as a side effect. Among the publications analyzed, oxidoreductases acting on CH-OH groups of donors (EC 1.1) have been the most studied, with high rates of successful reversals, while oxidoreductases acting on paired donors with incorporation or reduction of molecular oxygen (EC 1.14) have led to poor efficiencies, most probably due to the decoupling of flavin-dependent monooxygenases present in this group. In all studies covered by this review, positions were selected rationally and site directed mutagenesis was the most common methodology to introduce the changes. Moreover, when the enzyme structure was available and applied, better results have been obtained. Loop engineering provided the best results, however it was used in only 5 specific cases. We believe that more studies should consider this technique in the future, specifically to have a more compelling statement regarding the favorable results. NADP depending enzymes usually present positively charged residues in their coenzyme union site in positions able to interact with the phosphate group of the adenosine ribose moiety or to establish hydrogen bonding with it. Contrarily, NAD-dependent enzymes possess positively charged residues. These residues are the most added or eliminated depending on the desired coenzyme specificity. Although early works on coenzyme engineering already used enzyme structures for mutational design, the availability of novel structures crystalized with the corresponding coenzyme will undoubtedly help in future rational cofactor engineering. Moreover, most recent works include new computational strategies, which make coenzyme specificity changes much simpler. The satisfactory results obtained by using computational tools and algorithms make us believe that their application will be widespread in coenzyme engineering. We expect that improvement of these strategies, and also novel tools, will become available in the near future.

Despite we did not cover the natural reaction of the engineered enzymes, we believe it would be interesting in future coenzyme engineering studies to give an insight of the relation between the natural reaction of the enzyme, the test reaction reported and the success of the switch.

## Author contributions

All authors listed, have made substantial, direct and intellectual contribution to the work, and approved it for publication.

### Conflict of interest statement

The authors declare that the research was conducted in the absence of any commercial or financial relationships that could be construed as a potential conflict of interest.
